# Investigation on Household Medication-Taking Behavior and Affordability Among Patients Under Chronic Condition: A Survey Study in Gansu Province, China

**DOI:** 10.3389/fphar.2020.01280

**Published:** 2020-08-21

**Authors:** Xiaohui Wang, Ming Zhang, Shuaixiang Zhao, Meng Lv, Qinglin Li, Longjie He, Ming Hu

**Affiliations:** ^1^ School of Public Health, Lanzhou University, Lanzhou, China; ^2^ West China School of Pharmacy, Sichuan University, Chengdu, China

**Keywords:** chronic diseases, medication-taking behavior, household surveys, affordability, Gansu province

## Abstract

**Aim:**

This study aimed to investigate the household medication-taking behavior and affordability of patients with chronic diseases in Gansu Province. As well as to propose suggestions to assist improvement of related policies.

**Methods:**

A multistage stratified cluster sampling technique was used to select the research sites and households according to the WHO manual for the “Household Survey to Measure Access and Use of Medicines”. The impact factors of medication-taking behavior were estimated using binary logistic regression models. Medication treatment affordability of hypertension patients was evaluated.

**Results:**

A total of 1,080 completed questionnaires were collected, in which 690 families reported to have chronic patients in their home. The total number of chronic disease patients were 915. About 93% of the patient reported scheduled medicine intake. Approximately 86.60% of patients with chronic medical conditions were able to adhere to the physicians’ prescriptions. Age, gender, and the number of household chronic disease patients were the main factors influencing whether the patients take medication. Respondents’ self-reported monthly expenditure on household medicine accounted for 16.95% of their total monthly expenditure. The total cost of irbesartan and valsartan for hypertension treatment was two-fold higher than the minimum daily wage of the families.

**Conclusion:**

This study found that patients with chronic diseases have a high proportion of medication and medication compliance. However, family drug burden, especially for those living with hypertension are among the key challenges. Related policy needs to be revised to promote the affordability of medication under chronic conditions.

## Introduction

Non-communicable chronic diseases are a series of diseases with a long-term, intricate treatment, high disability rate, and high medical expenses (Xiang and Li, 2004). Chronic diseases pose a serious threat to human health and cause a considerable burden on the social economy. These diseases are common public health concerns and are one of the utmost threats worldwide (World Health Organization, 2000; World Health Organization, 2011; World Health Organization, 2013; Emmerick et al., 2015; Liao, 2018).

Instead of hospital treatment, most patients with chronic medical conditions generally prefer to take medication at home as prescribed by physicians. Rational use of medicines can effectively control the aggravation of chronic diseases and provide convenience in the treatment (Domingues et al., 2017). The potential danger and economic cost burden of household medicines have become the worldwide focus (Paniz et al., 2010; Tsiligianni et al., 2012; Li et al., 2016; Li et al., 2017). Household access to medicines for chronic diseases has been studied in several low-and middle-income countries in Africa and Latin America (Vialle-Valentin et al., 2012; Emmerick et al., 2015; Vialle-Valentin et al., 2015). The results showed that various parameters including patient’s age, family economic level, the education of the surveyed household head, and type of medical institution, affected the patient’s medication. The United States, United Kingdom, Japan, and other developed countries have conducted relevant studies on the storage and safe use of household medicines (LeBlanc and Choi, 2015; Kimura et al., 2018; Latif et al., 2018). The results showed that with the increase of the aging population there was an increment in the drug intake and risk of adverse drug reactions and interactions. Research on the use of household medicines in China has mainly been conducted in first-tier cities, such as Beijing, Shanghai and Chengdu, in a form of cross-sectional survey among patients with chronic diseases (Dong, 2012; Yu et al., 2016; Wang et al., 2019). The accessibility of household medicines has been good, but problems still exist, such as coverage of medical insurance and the use of antibiotics for acute diseases. These results showed that financial burden, medication safety with compliance, and the reserve of surplus drugs, are the main factors influencing medication usage behavior of chronic disease patients.

Medication expenditure usually account for an important part of healthcare expenditure. The medication affordability may affect the patient’s medication-taking behavior. The most common methods of measuring affordability are catastrophic health expenditure method, WHO/HAI standardized survey method, and the impoverishment method (Fang et al., 2016). Catastrophic health expenditure is based on the ratio of expenditure on household resources (Jan et al., 2018). The budget constraints are certain, excessive spending on a specific project will inevitably come at the expense of reducing other consumption, so when drug expenditure exceeds a certain percentage of household resources, it can be regarded as “catastrophic medication expenditure” or “not possible burden”. To measure catastrophic medication expenditure, a threshold must be determined. If the denominator is the total household expenditure, the commonly used threshold is 10%, that is, if the medication expenditure exceeds 10% of the total household expenditure, it is regarded as “catastrophic” (Guan et al., 2015).

Gansu Province is a landlocked province in Northwest China, which is one of the less developed provinces with the poorest health and economic status (the Per Capital Gross Regional Product ranked last but one in China in the year of 2018), and the least developed health care services in China (National Bureau of Statistics, 2019). The numbers of patients with chronic conditions have been increasing rapidly in recent years. The chronic disease prevalence rate in both urban and rural areas was higher than the national level in 2013. The prevalence rate of hypertension and diabetes in adults was 26.05% and 10.55%, respectively, which was slightly higher than the national chronic disease prevalence rate (25.15% and 9.65%) in 2013. The case fatality rate of chronic diseases in Gansu has increased from 72.60% in 1990 to 86.31% in 2015, and the burden of chronic diseases has accounted for 70.01% of the total disease burden (General Office of Gansu Provincial People’s Government, 2017). Recent studies showed that there is still an irrational prescription of antibiotics among primary health care workers and essential drugs list need to be improved in Gansu Province (Li et al., 2017). While, household medication-taking behavior and affordability for patients with chronic diseases in Gansu Province is still yet to know.

This study aimed to investigate household medication-taking behavior and the affordability for patients with chronic diseases in Gansu Province.

## Materials and Methods

### Study Design and Study Sites

This is a cross-sectional study conducted in Gansu Province during the spring of year 2018. The questionnaire was designed with reference to *WHO manual for the Household Survey to Measure Access and Use of Medicines* (Yu et al., 2016; World Health Organization, 2018). The domains of questionnaire mainly include the basic family situation, chronic disease patients’ number in this family, medication taking related behavior, and affordability.

A representative random sample was selected using a multistage stratified cluster sampling method combined with convenient sampling method. WHO manual recommends to select five regions to conduct the survey. In the first stage, the economic and geographical conditions of the cities/regions in Gansu Province were considered. Five cities, namely, Lanzhou(capital city), Wuwei, Zhangye, Dingxi, and Pingliang, were selected out of all the 14 cities. Followed by this, in each city, one municipal district (two in Lanzhou city since it is the capital city of Gansu Province with largest number of population) and two counties were selected. Then, in each county, two townships were chosen. Within each selected township, two villages were chosen according to the cooperation of local health care institutions and the households. Thirty households were then selected using convenient sampling approach at each sampled site as mentioned above. The household was the unit of interview, with a census achieved before the interview.

### Sample Size

The sample size (n=30 for each sampling unit) was adopted according to the WHO Manual for the Household Survey to Measure Access and Use of Medicines recommendation. Multistage stratified sampling approach was employed based on provincial socioeconomic status and other factors. In each of the five cities, 1 district (2 in Lanzhou) and 2 counties were sampled, and 2 townships in each county were sampled. Which end up with 6 districts, 10 counties, and 20 townships as our research sites (36 research sites in total). Then, convenience sampling was conducted at each interview site to select 30 potential interview households. A total of 1080 (36 sites * 30 household/site=1,080 households) completed questionnaires were collected, among which 690 households reported to have at least one family member with chronic condition (300 in urban and 390 in rural, respectively).

### Survey Procedures

The household survey was conducted from April–December, 2018. Eight student helpers were recruited. Before the fieldwork, a 2-days training for the student helpers was conducted to (1) familiarize them with the framework and concepts in the questionnaire; (2) review each question so the students understood the purpose of the questions; (3) discuss and introduce good interviewing skills; and (4) review data collection and recording procedures. A pilot survey was conducted in the communities outside the sampling area to improve the quality of the questionnaire. During the formal investigation, a group of two student helpers conducted household face-to-face interviews with the assistance of community health service centers, township health centers, and village clinics. After the interview, the questionnaires were collected on the spot.

### Drug Affordability Evaluation

Medication affordability was evaluated by using catastrophic health expenditure method and WHO/HAI standardized survey method. Catastrophic medication expenditure was evaluated by calculating the percentage of monthly medication expenditure over total household expenditure. The threshold was 10%. In the WHO/HAI standard treatment guidelines, the affordability assessment is based on the minimum daily salary standard for non-technical staff in government departments. In the 30-day treatment cycle for chronic diseases, the total cost required for the standard dose of the drug was compared. Affordability in this study is calculated using the following formula:

$$Total\;cost\;of\;medicine=\frac{\text{Median}\;\text{price}\;of\;drug/of\;package*DDD*treatment\;period}{\text{Minimum}\;\text{local}\;\text{daily}\;\text{salary}\;\text{standard}}\;$$

According to the equation, if the total cost of medicine is below onefold of the minimum daily salary, then the drug treatment plan is affordable (World Health Organization, 2003).

The median price of medicines came from the data of the Pharmaceutical Purchasing Office of the Gansu Provincial Public Resource Exchange Bureau, which has the median price of the drug unit of a certain specification and variety (Pharmaceutical Purchasing Department of Gansu Public Resources Trading Bureau, 2018). The DDD was based on the average daily dose prescribed in *the Pharmacopoeia of the People’s Republic of China and the Clinical Instructions*. The unloaded drugs were subjected to the daily dose limit specified in the drug label. The average daily dosage unit for adults was milligram, and the compound preparation was in the units of tablets. The minimum daily salary standard selected the average value of the minimum wage standard for 2018, announced by the Gansu Provincial People’s Government, which is 1,545 yuan per month or 51.5 yuan per day.

### Statistical Analysis

The completed questionnaires were collected and entered into Epidata 3.1 by the double-blind method and verified further. Data analysis was done using SPSS statistical software v.20 for Windows (IBM, Chicago, USA). Descriptive analyses were conducted to show the characteristic of the sample. Multivariate binary logistic regression models were used to explore the factors determining whether chronic diseases patients take medication or not. The proportion with 95% CIs for age, gender, and number of chronic disease patients in the family was reported.

## Results

### Basic Information of Sampled Household With Chronic Disease Patients

The majority of respondents were female (54.86%), aged from 45–74 years (62.31%). About 71% of the respondent were unemployed (category of “no job” accounted for a very small proportion of the total occupations, “farmer” and “no job” combined into the “unemployed” category). Most of the respondents (68.12%) hold a diploma of junior high school and below. 21.40% of the household reported has children over 5 years whereas 19.04% household has children under 5 years old. There were 74.71% families with senior citizens (over 60 years old). 58.34% of the households’ average monthly income was higher than 3,000 yuan in the urban area, while in rural area 77.18% was below 3,000 yuan. The majority of the household was covered by social medical insurance (98.26%). About fifty-two percentage of the oldest chronic disease patients were males in which 389 (56.38%) were over 65 years old (**Table 1**).

**Table 1:** Basic information of the respondents, households and chronic disease patients (n, %).

Group	Urban (n=300)	Rural (n=390)	Total (n=690)
**Respondents**			
Gender			
Male	132 (44.15)	261 (43.4)	311 (45.07)
Female	167 (55.85)	340 (56.6)	378 (54.86)
Age			
<44	89 (29.67)	141 (23.5)	156 (22.61)
45–59	65 (21.67)	249 (41.4)	220 (31.88)
60–74	79 (26.33)	166 (27.6)	210 (30.43)
>75	67 (22.33)	45 (7.5)	104 (15.07)
Educational level			
Junior high school and below	151 (50.33)	483 (80.4)	470 (68.12)
High school (higher vocational)	63 (21.00)	70 (11.6)	106 (15.36)
Bachelor (College)	84 (28.00)	107 (17.8)	110 (15.94)
Graduate and above	2 (0.67)	2 (0.30)	4 (0.58)
Working conditions			
Yes	120 (40.00)	146 (24.3)	203 (29.42)
No	180 (60.00)	455 (75.7)	487 (70.58)
**Sampled Households**			
Number of children			
>5 years old	62 (20.67)	154 (25.6)	147 (21.40)
≤5 years old	57 (19.00)	119 (19.8)	131 (19.04)
Number of the elderly	223 (74.33)	367 (61.1)	514 (74.71)
Monthly household incomes			
≤1,000	39 (13.00)	153 (39.23)	200 (28.99)
1,001~	85 (28.33)	148 (37.95)	233 (33.77)
3,001~	102 (34.00)	61 (15.64)	163 (23.62)
6,001~	68 (22.67)	21 (5.38)	89 (12.90)
>10,000	5 (1.67)	0 (0)	5 (0.72)
Self-reported family medical insurance			
Social medical insurance	293 (97.67)	385 (98.72)	678 (98.26)
Medical insurance for urban employees	116 (39.46)	17 (4.43)	133 (19.62)
Medical insurance for urban residents	177 (60.20)	20 (5.18)	197 (28.97)
New rural cooperative medical insurance	97 (33.11)	360 (93.26)	457 (67.30)
Business insurance	39 (13.00)	34 (8.72)	73 (10.58)
None	7 (2.33)	5 (1.28)	12 (1.74)
**Chronic disease patients**			
Gender			
Male	155(51.67)	203(52.05)	358(51.88)
Female	145(48.33)	187(47.95)	332(48.12)
Age			
<45	21(7.00)	12(3.08)	33(4.78)
45–65	104(34.67)	164(.42.05)	268(38.84)
>65	175(58.33)	214(54.88)	389(56.38)

### Chronic Diseases Conditions Among the Oldest Person in the Household

**Figure 1:**
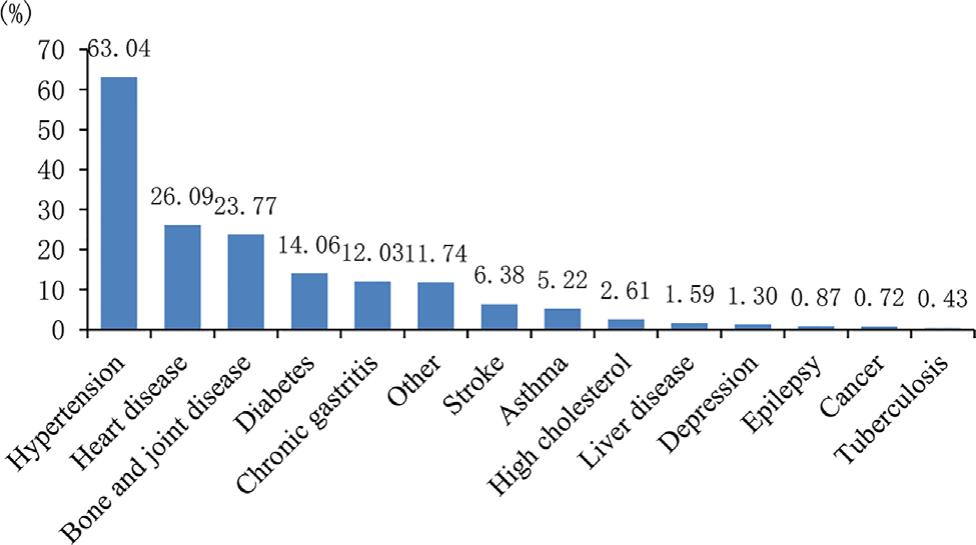
Chronic diseases condition distribution.

Hypertension, heart disease, bone and joint disease, diabetes, and chronic gastritis ranked the top five chronic conditions among the oldest people in 690 households. 435 senior citizens reported to have hypertension, which accounted for 63.04% of the chronic disease conditions among the oldest people (**Figure 1**).

### Chronic Disease Patients’ Medication-Taking Behavior

About 93% of the patient reported scheduled medicine intake. About eighty-eight percent of them reported that the medicine intake recommended by the physicians. Over ninety-three percent 86.60% of the patients who have taken medicine adhered to the physicians’ instruction. The most reported reason why the patients failed to take medicine as prescribed was because of the 58.14% alleviation of the symptoms. High prices and side effects were also identified as the reasons why the patients failed to take medicine (**Table 2**).

**Table 2:** Chronic disease patients’ medication-taking behavior and adherence (n, %).

Group	Urban (n=300)	Rural (n=390)	Total (n=690)
**Taken medication**			
Yes	283 (94.33)	359 (92.05)	642 (93.04)
Recommended by doctors	249 (87.99)	316 (88.02)	565 (88.01)
Patient himself	17 (6.01)	13 (3.62)	30 (4.67)
Recommended by neighbors	4 (1.41)	7 (1.95)	11 (1.71)
Recommended by other family members	5 (1.77)	4 (1.11)	9 (1.40)
No	17 (5.67)	31 (7.95)	48 (6.96)
**Adherence**			
Yes	253(89.40)	303(84.40)	556(86.60)
No	30(10.60)	56(15.60)	86(13.40)
**Reasons for failure to adhere**			
Getting better	16(53.33)	34(60.71)	50(58.14)
High prices	7(23.33)	10(17.86)	17(19.77)
Side effects	7(23.33)	7(12.50)	14(16.28)
Unacceptable way and taste of medicines	4(13.33)	7(12.50)	11(12.80)
Far distance of the drug stores	1(3.33)	6(10.71)	7(8.14)
Not available	2(6.67)	2(3.57)	4(4.65)

### Factors Influence Medication-Taking Behavior

The result of binary logistic regression shows that age, sex, and the number of chronic disease patients in the family were the factors influencing the medication intake of chronic diseases patients. Men with chronic diseases were significantly less likely to take medication than women (OR=0.33, 95%CI=0.17–0.64, P<0.05). Chronic illness patients of age <45 years were significantly less likely to take medication compared to the patient aged 65 and above (OR=0.19, 95%CI=0.07–0.57, P<0.05). Families with more than one member under chronic illness condition were significantly less likely to take medication according to the prescription (OR=0.37, 95% CI=0.16–0.84, P<0.05, **Table 3**).

**Table 3:** Factors influence medication-taking behavior.

Group	β	SE	Wald	*P*	OR	OR (95%CI)
Male(vs Female)	-1.112	0.340	10.704	0.001*	0.329	0.169–0.640
Age(years)						
Under 45(vs over 65)	-1.646	0.549	8.981	0.003*	0.193	0.066–0.566
45–65(vs over 65)	-0.411	0.335	1.506	0.220	0.663	0.344–1.278
Family peoples with exceeds 2	0.310	0.336	0.851	0.356	1.364	0.705–2.637
Families with more than one member under chronic illness condition	-0.997	0.417	5.727	0.017*	0.369	0.163–0.835
Urban residents	0.449	0.326	1.900	0.168	1.566	0.828–2.964
Arrived at the nearest public medical institution time under 15 min	0.505	0.317	2.531	0.112	1.656	0.890–3.084
Constant	3.625	0.545	44.293	0.000	37.527	

*P < 0.05. Bold indicates difference with an α level of less than 0.05.

### Affordability Analysis

Monthly average expenditure on family medicines was 359.57 yuan, thereby accounting for 16.95% of the total monthly household expenditure. The proportion of rural household medicine expenditure was 2.75 percent higher than the urban household. 84.78% of the families reported affordability of all the required medicine. 28.99% of the families with chronic disease patients responded that they might tend to purchase more medicine if covered by medical insurance [Affordability of chronic diseases (n, %) (**Table 4** **)]**.

**Table 4:** Affordability of chronic diseases (n, %).

Group	Urban (n=300)	Rural (n=390)	Total (n=690)
Average monthly household expenditure of medicines (yuan)	429.06	305.31	359.57
Average monthly household expenditure on health (yuan)	783.38	408.57	572.89
Monthly medication expenditures account for total household expenditure (%)	15.74	18.49	16.95
Families can fully fund all required medications	263 (87.67)	322 (82.56)	585 (84.78)
Buying medical insurance will increase the use of medicines	45 (15.00)	135 (34.62)	200 (28.99)

### Commonly Used Medicines and Affordability Among Hypertension Patients

Since hypertension ranked the first condition among our respondent households, information on the most commonly used medication and its affordability for hypertension patients was collected and analyzed. As shown in **Table 5**, 11 medications were identified as commonly used medicine. The top five drugs were nifedipine, danshen (a traditional Chinese medicine) tablets, bendazol hydrochlorothiazide, xaptopril and metoprolol tartrate. Nine out of the eleven drugs were on the national essential drug list. **Table 6** showing the total cost of the two treatment periods of Irbesartan and Valsartan was higher than the minimum daily salary of one time, thus reflecting its poor affordability.

**Table 5:** Number of commonly used drugs and cases in patients with hypertension.

Drug name	Dosage forms	Number of uses	Essential medicines
Nifedipine	Tablet, sustained release tablet	56	Yes
Danshen Tablets	Tablet, granule, capsule, pill	56	Yes
Bendazol Hydrochlorothiazide	Capsule	55	No
Captopril	Tablet	52	Yes
Metoprolol tartrate	Tablet	46	Yes
Aspirin	Tablet, enteric tablet	33	Yes
Irbesartan	Tablet	26	No
Indapamide	Tablet, sustained release tablet	20	Yes
Nitrendipine	Tablet	19	Yes
Valsartan	Capsule	17	Yes
Reserpine	Tablet	16	Yes

**Table 6:** Affordability of hypertension treatment drug for investigated residents.

**Generic name of drug**	**Specification**	**DDD**	**Median price (yuan)**	**Equivalent to minimum daily salary multiple**
Reserpine	100 tablets	1 tablet	8.74	0.05
Nifedipine	10 mg×100 tablets	30 mg	5.02	0.09
Captopril	25 mg×100 tablets	50 mg	7.39	0.09
Nitrendipine	10 mg×100 tablets	20 mg	9.67	0.11
Indapamide	2.5 mg×30 tablets	2.5 mg	14.07	0.27
Bendazol hydrochlorothiazide	48 capsules	3 capsules	9.65	0.35
Metoprolol tartrate	25 mg×20 tablets	150 mg	5.09	0.89
Irbesartan	150 mg×7 tablets	150 mg	17.85	1.49
Valsartan	80 mg×7 tablets	80 mg	35.18	2.93

## Discussion

This study explored household medication-taking behavior and the affordability of patients with chronic diseases in Gansu Province. The current analysis found that 93.04% of the patients with chronic diseases reported having taken medicines, and the vast majority of the medication they took was recommended by health care providers. The patient’s gender and age affect whether they take medication. The catastrophic health care payment method and the WHO/HAI standard survey method were used for affordability evaluation. Monthly drug expenditure of the total household expenditure was more than 10%, and can be considered as a “catastrophic” drug expenditure for the household. Since hypertension accounted for the largest proportion of our respondent household, commonly used drug affordability for patients with hypertension was investigated. The result showed that the total cost of Angiotensin Receptor Blocker (ARB) drugs such as irbesartan and valsartan is less affordable.

The first objective of this study was to investigate household medication-taking behavior. This including whether the patient have taken medication, what medication did he/she take, who recommended the drugs for them and whether they took the medication adhere to the doctors` recommendation. One of the most important concepts is the rational use of medicines/reasonable medication. According to WHO (World Health Organization, 1987), rational use of medicines requires that patients receive medications based on their clinical needs, in doses limits to meet the requirements, for an adequate period, and at the lowest possible cost for patients and their community. It can improve the patient’s prognosis and minimize drug-related side effects. Patients with chronic diseases usually need long-term or even lifelong medication to control the alleviation of condition. Unreasonable drug treatment is the leading cause for mortality (Ramia et al., 2017). The current study found that patients over 65 years old accounted for 56.38%. Among all of the chronic patients, 93.04% of them reported to have taken medicines, and the vast majority of the medication they took was recommended by health care providers (88.01%). The patient’s gender and age also affect whether they take medication. Female patients tend to take more drugs than males in our survey, similar to the results of Anita K. Wagner et al. conducted in 53 countries (Wagner et al., 2013). The reasons might be the following: first, women are more sensitive and care about their health; second, as caregivers, they have more opportunities to acquire the knowledge of disease and have more time to receive treatment. Patients aged 65 and above are more likely to take medicine than those under the age of 45. The elderly is prone to medication-related side effects due to comorbidities, multi-drug combination, and long-term medication.

Adherence implies the active involvement of patients in the medication-taking process (World Health Organization, 2003). Our survey showed that 86.60% of the patients adhered to the physician’s instruction in medication-taking process. Regarding to the reasons why patients did not take medication as advised was “feel getting better”, followed by the “high price” of the medicine, and “worried about side effects”. Poor adherence to prescription medications might undermine the effectiveness of treatment, increase the risk of adverse health outcomes, reduce the quality of life, increase the number of visits and hospitalization, and result in increased medical expenditure as well as mortality (Chisholm-Burns and Spivey, 2012).

Pellegrino et al. (2009) reported that less than half of the chronic patients were still able to adhere to the prescription after 12 months’ drug treatment. The number of deaths caused by poor compliance were about 125,000 per year. Previous studies showed that adherence to prescriptions among elderly patients with a chronic condition is poor (Chen et al., 2007). Hegde SK’s (Hegde et al., 2016) research on elderly patients with chronic diseases in India found that 63.60% of the people took the medicine completely as directed by the doctor. Enthusiastic doctor-patient communication, the doctor’s interpretation of the consequences of not taking medicine, and the side effect of the medicine are the key determinants of whether the patients can continue to take the medicine. Therefore, our research implies the requisite of strengthen guidance on the safe use of drugs among elderly patients. Community-based advocating on the correct use of commonly used drugs, response to the side effect, and storage method of family medicines should be offered (Lin et al., 2017). From the healthcare worker’s side, it is necessary to communicate with patients during the prescription, so that patients can fully understand the importance of adherence and improve their awareness, self-management ability, and compliance.

As far as the affordability of medicines is concerned, price is the first consideration (Helfer et al., 2012; Washington, 2018). Our study used to measure the affordability of medicines, namely, the catastrophic expenditure evaluation method and WHO/HAI standard survey method. 84.78% of households with chronic diseases interviewed can fully afford medical expenses. Household monthly drug expenditure accounts for 16.95% of the total expenditure which exceeds 10% of the total household expenditure, it is regarded as “catastrophic”. The proportion is also higher than that of in Beijing and Sichuan province in China (Deng, 2018; Deng et al., 2018). From a national perspective, the overall affordability of household drugs in Gansu Province needs to be improved.

This survey found that the proportion of hypertensive patients was the highest, and the monthly drug expenditure of patients’ families accounted for 16.38% of the total expenditure. So, the drug affordability of this group using the WHO/HAI standard survey method was evaluated. Compound reserpine, nifedipine, and captopril are among the top three in terms of affordability, similar to the results of Dou and Wang (2017) survey on oral antihypertensive drugs in five public medical institutions in Luohe City, China. As a first-line drug for hypertension, ARB drugs have the advantages of good therapeutic effect, less side effects, and effective prevention of complications (Guo, 2019). However, this survey shows that the total cost of ARB drugs such as irbesartan and valsartan is more than double the average minimum daily wage. This indicates the relatively poor affordability of such drugs in Gansu Province The results showed that combined drug treatment accounted for 75.71%, of which the calcium antagonist nifedipine was used the most, and was in line with the calcium antagonist-based combined treatment plan in the National Guidelines for Rational Use of Hypertension, and was the optimal antihypertensive plan for hypertension patients in China (National Health and Family Planning Commission, 2017).

Essential medicines are those that satisfy the priority health care needs of the population. They are selected with due regard to disease prevalence and public health relevance, evidence of clinical efficacy and safety, comparative costs, and cost-effectiveness (World Health Organization, 2017). Medicine therapy is one of the main treatments for hypertension. To release the financial burden of patients, the essential medicines list is an important policy approach (Mendis et al., 2007; Vialle-Valentin et al., 2015; Wang et al., 2017). The policy intended to introduce the drugs in the appropriate dosage forms, with assured quality, and at a price the individual and the community can afford. In the context of our interview, most of the commonly used medicine in hypertensive residents is in the essential drug list. But exceptions still exist, like compound Debazole Hydrochlorothiazide and irbesartan, the two are not on the national essential medicine list. Bendazol hydrochlorothiazide capsule is a conventional and effective antihypertensive compound. Irbesartan is an ARB, not only has a good antihypertensive effect, but can also reduce the probability of cardiovascular events in patients with hypertension. In addition, no Calcium Channel Blocker (CCB) common adverse reactions such as ankle edema were reported in Irbesartan treatment (Sun et al., 2011). Developed cities in China like Shanghai (Shen et al., 2018), has included Irbesartan on the essential drug list. But there is still a long way to go for the less developed area such as north-western China. It is recommended that policymakers should include factors like safety, effectiveness, and economy into consideration, to comprehensively evaluate the burden of chronic disease as well as social and economic benefits for chronic disease prevention and control. These factors assist in optimizing essential medicine list to improve reasonable medication in chronic disease patients.

This study is the first household face-to-face survey emphasizing the explored medication-taking behavior and the affordability in patients with chronic diseases in less developed area of China. However, there are several limitations in the study. First, as a cross-sectional study, it prevented us from investigating the causal relationship between socioeconomic status and medication-taking behavior, the direction of causality cannot be established. Second, the sample was limited in terms of location and number of households interviewed. When choosing the household, a convenient sampling approach was used according to the cooperation of the household, which might induce selection bias. Last but not least, our findings can only represent the situation in Gansu Province, it cannot be generalized to represent the whole country.

## Conclusion

This study found that patients with chronic diseases have a high proportion of medication and medication compliance. However, family drug burden, especially for those living with hypertension are among the key challenges. Related policy needs to be revised to improve the affordability of medication under chronic conditions.

## Data Availability Statement

The raw data supporting the conclusions of this article will be made available by the authors, without undue reservation, to any qualified researcher.

## Ethics Statement

The studies involving human participants were reviewed and approved by School of Public Health, Lanzhou University Ethics Committee. The participants provided their written informed consent to participate in this study.

## Author Contributions

XW arranged field work and responsible for manuscript writing. MH put forward constructive comments on the article. MZ is responsible for data analysis and writing. ML, QL, and LH are responsible for data collection and screening. SZ has made precise revisions to the full text.

## Funding

The work was supported by the National Natural Science Foundation of China (grant number 71473170).

## Conflict of Interest

The authors declare that the research was conducted in the absence of any commercial or financial relationships that could be construed as a potential conflict of interest.
