# Supplementary material for: Investigation on Household Medication-Taking Behavior and Affordability Among Patients Under Chronic Condition: A Survey Study in Gansu Province, China
Source: Front Pharmacol. 2020 Aug 21;11:1280. doi: 10.3389/fphar.2020.01280 (PMC7472568; doi:10.3389/fphar.2020.01280)
Supplement: Supplementary file 1 [file Table_1.docx]

**Supplementary material**

| **Table1. The lists of cities, districts, counties and townships surveyed in Gansu, China(location, number of household interviewed)** | | | |
| --- | --- | --- | --- |
| City | District/County | Township | Total |
| Lanzhou | Anning District (30) | - | 239 |
|  | Xigu District (30) | - |  |
|  | Gaolan County (30) | Xicha（30） |  |
|  |  | Heishi（30） |  |
|  | Yuzhong County(30) | Gancao（30） |  |
|  |  | Lianda(29) |  |
| Wuwei | Liangzhou District (30) | - | 207 |
|  | Minqin County (27) | Suwu (30) |  |
|  |  | Quanshan (30) |  |
|  | Gulang County (31) | Fengquan (30) |  |
|  |  | Gufeng (29) |  |
| Zhangye | Ganzhou District (30) | - |  |
|  | Gaotai County (30) | Heli (30) | 210 |
|  |  | Hangdao (30) |  |
|  | Shandan County (30) | Chenhu (30) |  |
|  |  | Weiqi (30) |  |
| Dingxi | Anding District (30) | - | 211 |
|  | Tongwei County (30) | Xiangnan (30) |  |
|  |  | Biyu (30) |  |
|  | Weiyuan County (30) | Qingping (30) |  |
|  |  | Qiaoyu(31) |  |
| Pingliang | Kongtong District (30) | - | 213 |
|  | Jingning County (31) | Siqiao (31) |  |
|  |  | Chengchuan (31) |  |
|  | Jinchuan County (30) | Libao (30) |  |
|  |  | Yudu (30) |  |
| Total sample | 479 | 601 | 1080 |
